# The biological activity of serum bacterial lipopolysaccharides associates with disease activity and likelihood of achieving remission in patients with rheumatoid arthritis

**DOI:** 10.1186/s13075-022-02946-z

**Published:** 2022-11-21

**Authors:** J. Parantainen, G. Barreto, R. Koivuniemi, H. Kautiainen, D. Nordström, E. Moilanen, M. Hämäläinen, M. Leirisalo-Repo, K. Nurmi, K. K. Eklund

**Affiliations:** 1 Translational Immunology Research Program, Faculty of Medicine, University of Helsinki, PL 4 (Yliopistonkatu 3), 00014 Helsinki, Finland; 2 Orton Orthopedic Hospital, Helsinki, Finland; 3 Department of Rheumatology, Inflammation Center, University of Helsinki and Helsinki University Hospital, Helsinki, Finland; 4 Kanta-Häme Central Hospital, Riihimäki, Finland; 5 Folkhälsan Research Center, Helsinki, Finland; Unit of Primary Health Care, Kuopio University Hospital, Kuopio, Finland; 6 Department of Internal medicine and rehabilitation, Helsinki University and Helsinki University Hospital, Helsinki, Finland; 7 The Immunopharmacology Research Group, Faculty of Medicine and Health Technology, University of Tampere and Tampere University Hospital, Tampere, Finland

**Keywords:** Rheumatoid arthritis, Lipopolysaccharides, Dysbiosis

## Abstract

**Background:**

Dysbiotic intestinal and oral microbiota have been implicated in the pathogenesis of rheumatoid arthritis (RA), but the mechanisms how microbiota could impact disease activity have remained elusive. The aim of this study was to assess the association of the biological activity of serum lipopolysaccharides (LPS) with disease activity and likelihood of achieving remission in RA patients.

**Methods:**

We measured Toll-like receptor (TLR) 4-stimulating activity of sera of 58 RA patients with a reporter cell line engineered to produce secreted alkaline phosphatase in response to TLR4 stimulation. Levels of LPS-binding protein, CD14, and CD163 were determined by ELISA assays.

**Results:**

The patient serum-induced TLR4 activation (biological activity of LPS) was significantly associated with inflammatory parameters and body mass index at baseline and at 12 months and with disease activity (DAS28-CRP, *p*<0.001) at 12 months. Importantly, baseline LPS bioactivity correlated with disease activity (*p*=0.031) and, in 28 early RA patients, the likelihood of achieving remission at 12 months (*p*=0.009). The level of LPS bioactivity was similar at baseline and 12-month visits, suggesting that LPS bioactivity is an independent patient-related factor. Neutralization of LPS in serum by polymyxin B abrogated the TLR4 signaling, suggesting that LPS was the major contributor to TLR4 activation.

**Conclusion:**

We describe a novel approach to study the biological activity of serum LPS and their impact in diseases. The results suggest that LPS contribute to the inflammatory burden and disease activity on patients with RA and that serum-induced TLR4 activation assays can serve as an independent prognostic factor.

**Graphical Abstract:**

A graphical summary of the conclusions of the study.
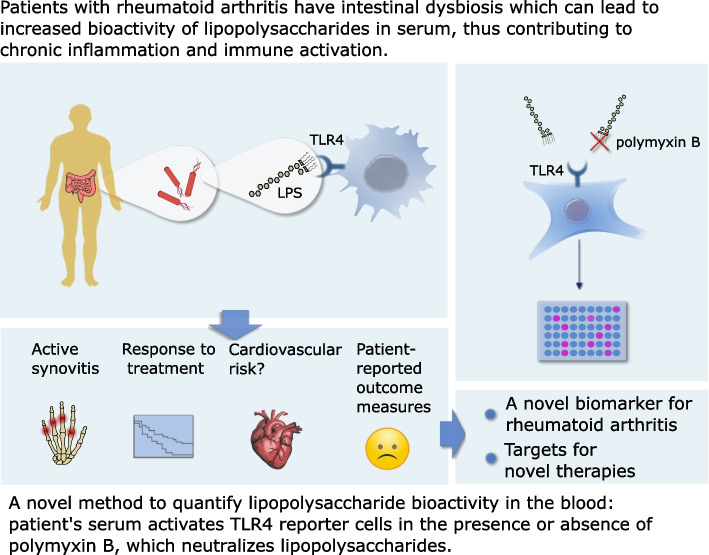

**Supplementary Information:**

The online version contains supplementary material available at 10.1186/s13075-022-02946-z.

## Introduction

Recent studies have implicated intestinal and oral bacteria in the pathogenesis of rheumatoid arthritis (RA) [1]. Various pathogen-associated molecular patterns (PAMPs) derived from the intestinal bacteria have a potential to activate the immune system, thus contributing to the inflammatory burden, but they also potentially drive the autoimmune process by providing the second signal for T cell activation [2]. An autoimmune reaction against citrullinated proteins has been suggested to emerge at the mucosal surfaces of the oral cavity or the lung where citrullination of proteins by *Porphyromonas gingivalis* and *Aggregatibacter actinomycetemcomitans* could initiate the generation of anti-citrullinated protein autoantibodies (ACPAs) [3, 4]. *P. gingivalis* colonization did not correlate with RA-specific antibodies or disease status in some recent studies, however [5].

Disturbance of intestinal permeability or dysbiosis of the intestinal microbiota can increase the release of PAMPs from the gut into the systemic circulation in RA [6]. RA patients have increased serum levels of zonulin, which is a biomarker and possible mediator of increased intestinal permeability [7]. Lipopolysaccharides (LPS), an integral part of the outer membrane of gram-negative bacteria, are some of the most extensively studied PAMPs. Binding of LPS to the Toll-like receptor (TLR) 4 on the cell surface activates a signaling cascade leading to the activation of nuclear factor (NF)-κB signaling with ensuing expression of myriad of pro-inflammatory mediators [8]. TLR4 recognition of LPS requires facilitation from the LPS-binding protein (LBP) and co-receptors cluster of differentiation (CD)14 and myeloid differentiation (MD)2 [9]. LBP is secreted primarily from hepatocytes and intestinal cells, and it is widely considered a surrogate marker for serum LPS levels [10, 11].

Several lines of evidence suggest a role for LPS in pathogenesis of RA. In patients with RA, TLR4 is abundantly expressed in the inflamed synovial tissue, and levels of antimicrobial response factors are elevated in patient serum [12, 13]. In a collagen-induced mouse model of arthritis, an LPS injection accelerated the onset of arthritis and led into its reactivation [14, 15]. Also, a number of studies have reported intestinal dysbiosis in RA, and patients with recent onset RA have been shown to have lower abundance of the *Bifidobacterium* and *Bacteroides* families, higher abundance of *Prevotella copri*, and decreased gut microbial diversity [16–18].

Difficulties in reliable quantification of LPS have hindered studies on the role of LPS in human diseases [19]. Serum LPS concentration does not necessarily reflect the pro-inflammatory potential of the LPS, as the ability of LPS derived from different bacteria to activate TLR4 vary significantly; some types of LPS can even reduce the immune activation by inducing tolerance [20, 21]. Surrogate markers of LPS such as LBP, CD14, or CD163 have been utilized to overcome these problems [19, 22]. CD163 is a transmembrane protein on monocytes and macrophages, the expression and release of which is induced by LPS [23].

We hypothesize that bacterial LPS contribute significantly to the inflammatory burden in RA and can modulate the disease activity. To study the level of biologically active circulating LPS in RA patients, we measured the TLR4-activating potential of the sera of RA patients and studied its correlation with disease activity and inflammatory markers.

## Patients and methods

### Patients

Sixty female RA patients of White ethnicity were recruited prospectively from the Helsinki University Hospital Department of Rheumatology. All new onset RA patients and patients with poor response to RA treatment who did not meet the exclusion criteria were enrolled. The number of patients enrolled was based on the design of a previous study based on the cohort [24]. RA patients fulfilled the European Alliance of Associations for Rheumatology/American College of Rheumatology (EULAR/ACR) 2010 classification criteria for RA [25]. Of the patients, 58 were re-examined at a follow-up visit after 12 months and included in the study; 30 patients had previously untreated early RA (ERA) and started disease-modifying anti-rheumatic drugs (DMARDs), and 28 had chronic RA (CRA) with inadequate response to conventional synthetic DMARDs (csDMARDs). First-line treatment for ERA patients was in general a combination of csDMARDs, typically methotrexate (Mtx), sulphasalazine, and hydroxychloroquine. Half of the CRA patients used a combination of csDMARDs, and their treatment was reinforced with a biological DMARD, most often a tumor necrosis factor inhibitor. The exclusion criteria included pre-existing cardiac or renal disease and conventional cardiac risk factors such as male sex, age over 70, current smoking, and diabetes.

Remaining sample material was insufficient for 3 patients at baseline and 2 patients at the 12-month follow-up, and sera were thus available for 55 patients at baseline and for 56 patients at follow-up. Clinical disease activity was recorded based on the disease activity score (DAS28-CRP) supplemented by self-reported pain, patient global assessment (visual analog scale), and Health Assessment Questionnaire (HAQ) results. Disease Activity Indexes (DAI) could not be calculated as data on physician global assessment was lacking. Disease remission was defined according to the ACR/EULAR 2011 boolean-based definition [26]. Between the two visits, DAS28-CRP decreased significantly in both groups, but the inflammatory markers decreased more significantly in the ERA group [24].

### Biochemical analyses

We quantified TLR4 activation representing LPS bioactivity from serum samples with HEK-Blue^TM^ hTLR4 reporter cells (InvivoGen, San Diego, CA, USA) engineered to produce secreted alkaline phosphatase (SEAP) in response to TLR4 stimulation. The cells were cultured following the manufacturer’s protocol in Dulbecco’s modified Eagle’s medium without phenol red supplemented with penicillin-streptomycin, L-glutamine, and 10% (v/v) fetal bovine serum. We seeded 50,000 cells per well on a 96-well cell culture plate in 180 μl of cell culture media. Serum samples were diluted 5-fold in endotoxin-free water (bioMérieux Industry, Starnberger See, Germany) to obtain a final working concentration of 2% (v/v). We then added 20 μl of 5-fold diluted serum sample in duplicates to each well for a final working volume of 200 μl. TLR4-signaling inhibitor CLI-095 1 μg/ml (InvivoGen) was applied to control for any non-TLR4 mediated SEAP secretion. To determine whether TLR4 activation was due to LPS, we added another set of sample duplicates with 0.1 mg/ml of polymyxin B (Sigma-Aldrich, Buchs, Switzerland), an LPS inhibitor. Human AB serum 2% (v/v, Sigma-Aldrich) served as a negative control. A set of standard dilutions was created with LPS-B5 Ultrapure from *Eschericia coli O55:B5* (InvivoGen) diluted in endotoxin-free water with 2% Human AB serum. After incubation in 37° C for 24 h, we collected cell culture media from the wells by aspiration.

For the QUANTI-Blue detection for SEAP, 20 μl of media sample per well was pipetted on 96-well plates and subsequently 180 μl of QUANTI-Blue^TM^ Solution (InvivoGen), prepared following the manufacturer’s instructions, was added to the wells. Optical densities (OD) at 630 nm were read with a microplate reader (FLUOstar Omega, BMG Labtech, Ortenberg, Germany) after incubating the plates for 6 h in 37°C. To correct for possible bias caused by slight variations in the temperature of the plates during incubation, we included a negative human AB serum control for each sample on the plates and subtracted the control OD from the sample readings. TLR4 activation due to LPS was then determined by subtracting the activity remaining in the presence of polymyxin B from the total TLR4 activation measured. A linear standard curve was plotted.

Human CD163 and CD14 concentrations were measured by respective Quantikine ELISA Immunoassays (R&D Systems, Minneapolis, MN, USA) according to the manufacturer’s protocol. Serum samples were diluted 100-fold for CD163 and 1000-fold for CD14 assays. Human LBP concentration was quantified by a DuoSet ELISA Immunoassay (R&D Systems) with serum samples diluted 1000-fold. ODs were measured with the microplate reader set to read the absorbance at 450 nm with a wavelength correction set at 540 nm. A standard curve was plotted and a polynomial best fit curve was constructed based on a standard dilution series after subtracting the zero standard OD. LPS concentrations in the sera were also measured with an EndoLISA assay (bioMérieux) after 100-fold dilution of the sera in endotoxin-free water and heat treatment at 75°C for 15 min. After a binding step of 18.5 h, fluorescence readings were obtained with a microplate reader (Spark, Tecan Austria GmbH, Grödig, Austria).

Concentrations of high-sensitivity CRP (hsCRP), serum amyloid A (SAA), glycoprotein YKL-40, E-selectin, resistin, visfatin, and interleukin (IL)-6 were measured with enzyme-linked immunosorbent assays (ELISA) as described in a previous publication [24]. Total cholesterol, high-density lipoprotein (HDL) cholesterol, low-density lipoprotein (LDL) cholesterol, and triglycerides (TG) were measured by the hospital laboratory (HUSLAB clinical laboratory of Helsinki University) using accredited methods according to ISO 17025 and 15189 standards (FINAS). Patient body composition was measured by bioelectrical impedance analysis with a Salter scale model 9106 (Salter housewares, Manchester, UK).

### Statistical analyses

Statistical analyses were undertaken with Stata software version 17.0 (StataCorp, College Station, TX, USA) and SPSS version 28 (SPSS, Inc., Chicago, IL, USA). Statistical analyses were 2-sided, and differences between groups were considered statistically significant at *p*≤0.05. Spearman correlation coefficients were calculated for correlations of continuous variables between groups, and 95% confidence intervals were estimated based on Fisher’s r-to-z transformation. Differences between groups were compared by the Fisher-Pitman permutation test. Differences in paired samples between groups were compared by the Wilcoxon signed rank test. Differences between classes of categorical variables were tested with cross-tabulation and *χ*^2^ test. Patients with missing data were included in the analyses whenever possible. Principal component analyses were performed with the *stats* package (version 4.0.2) in R after log(x+1) transformation of the data. Correlation plots were created with the *corrplot* package (version 0.84) in R.

## Results

### Patient characteristics

Koivuniemi et al. [24] have previously described the patient population comprising of 58 female RA patients. Their clinical characteristics are shown in supplementary table 1. Of the 58 patients, 30 had ERA and were treatment-naïve, whereas 28 patients had CRA with inadequate response to conventional synthetic disease-modifying anti-rheumatic drugs, and their medication was modified at the baseline. The follow-up visit took place 12 months later. Between the two visits, DAS28-CRP decreased significantly in both groups [24]. Based on active disease at the baseline, the analyses concern both ERA and CRA patients unless otherwise indicated. Missing data is presented in supplementary table 2.

### Serum LPS bioactivity at baseline associates with inflammatory parameters and body mass index (BMI)

First, we explored for any correlations at baseline between serum-induced TLR4 signaling in the reporter cells (LPS bioactivity), inflammatory parameters, and disease activity. No correlation was found between LPS bioactivity and baseline disease activity as measured by DAS28-CRP (*r*=+0.13 [95% confidence interval (CI) −0.15 to +0.39], *p*=0.35) or the number of swollen (*r*=−0.02 [95% CI −0.29 to +0.25], *p*=0.88) or tender joints (*r*=−0.05 [95% CI −0.23 to +0.34], *p*=0.70). LPS bioactivity correlated with the inflammatory parameters erythrocyte sedimentation rate (ESR, *r*=+0.28 [95% CI +0.01 to 0.52], *p*=0.037), SAA (*r*=+0.35 [95% CI +0.09 to 0.57], *p*=0.008), YKL40 (*r*=+0.27 [95% CI +0.002 to 0.51], *p*=0.042), and E-selectin (*r*=+0.33 [95% CI +0.07 to 0.56], *p*=0.013), but not with hsCRP, resistin, visfatin, or IL-6 (Supplementary table 3a). Baseline LPS bioactivity correlated also with BMI (*r*=+0.42 [95% CI +0.17 to 0.62], *p*=0.002), the amount of adipose tissue (*r*=+0.47 [95% CI +0.21 to 0.67], *p*<0.001), blood pressure (systolic *r*=+0.40 [95% CI +0.14 to 0.61], *p*=0.002 and diastolic, *r*=+0.37 [95% CI +0.14 to 0.61], *p*=0.006), and advancing age (*r*=+0.45 [95% CI +0.21 to 0.64], *p*<0.001) (Supplementary table 3a), but not with the presence of ACPAs (mean±SD [standard deviation] 0.24±0.12 vs mean±SD 0.19±0.08 EU/ml, *p*=0.22).

As previous studies have utilized LBP, CD14, and CD163 as surrogate markers for LPS levels, we analyzed their correlations with LPS bioactivity. LPS bioactivity at baseline correlated significantly with the CD163 (*r*=+0.44 [95% CI +0.19 to 0.63], *p*<0.001), but not with LBP (*r*=+0.22 [95% CI −0.06 to +0.46], *p*=0.12) or CD14 (*r*=+0.06 [95% CI −0.19 to +0.35], *p*=0.53). Baseline LBP correlated with DAS28-CRP (*r*=+0.39 [95% CI +0.13 to 0.60], *p*=0.004), ESR (*r*=+0.47 [95% CI +0.22 to 0.66], *p*<0.001), and hsCRP (*r*=+0.47 [95% CI +0.22 to 0.66], *p*<0.001) (Supplementary table 3b). CD163 correlated also with the markers of inflammation and with disease activity (Supplementary table 3c). Furthermore, baseline LBP and CD163 correlated with each other (*r*=+0.50 [95% CI +0.26 to 0.68], *p*<0.001). CD14 correlated with LBP, CD163, and ESR, but not with disease activity (Supplementary table 3d). The total serum LPS concentrations as measured by the EndoLISA assay did not correlate with disease activity, inflammatory parameters, and had a significant correlation at the baseline with CD14 and CD163 but not LPS bioactivity or LBP (Supplementary table 3e).

### Serum LPS bioactivity at the follow-up visit associates with inflammatory parameters and disease activity

**Fig. 1:**
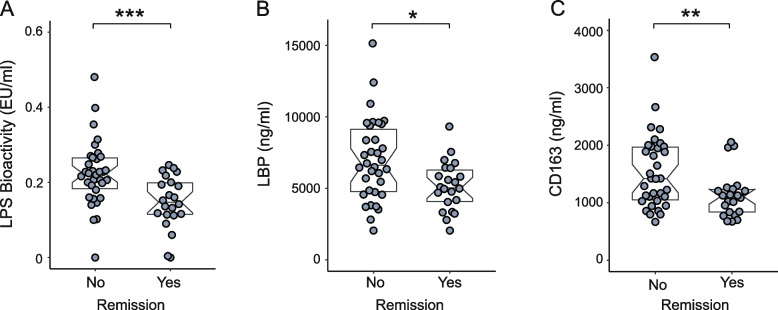
Remission (ACR/EULAR 2011) of rheumatoid arthritis is associated with lower levels of serum LPS bioactivity (**A**), LBP (**B**), and CD163 (**C**) concentrations at the 12-month follow-up visit. Notched boxplots represent interquartile ranges and 95% confidence intervals of the medians. *p≤0.05, **p≤0.01, ***p≤0.001

**Fig. 2:**
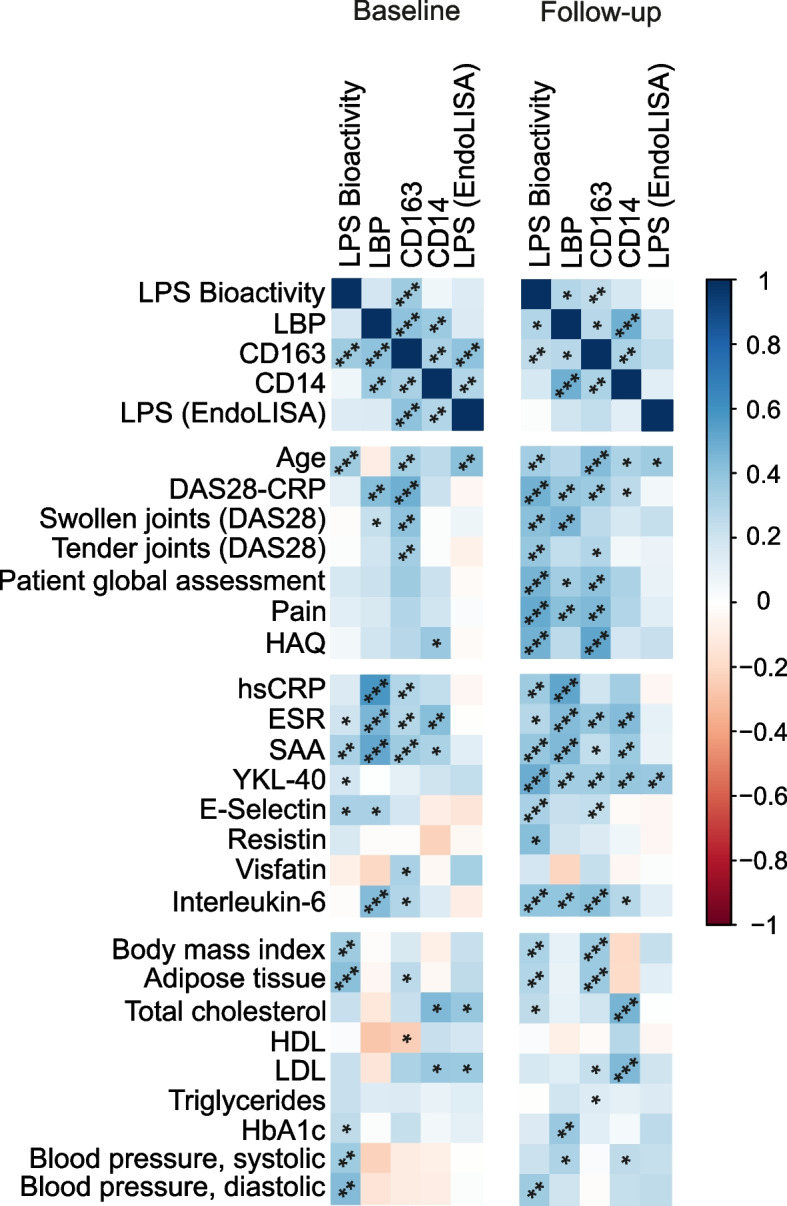
A correlation plot of serum LPS bioactivity and the concentrations of LBP, CD163, CD14, and LPS (EndoLISA) with each other, disease activity, metabolic factors, and inflammatory biomarkers. Colors represent Spearman correlation coefficients. *p≤0.05, **p≤0.01, ***p≤0.001

At the follow-up visit after 12 months, the correlations between LPS bioactivity and disease activity and hsCRP became statistically significant (for DAS28-CRP *r*=+0.48 [95% CI +0.24 to 0.67] and *p*<0.001, for hsCRP *r*=+0.41 [95% CI +0.15 to 0.61] and *p*=0.004, and for ESR *r*=+0.29 [95% CI +0.02 to 0.52] and *p*=0.030) (Supplementary table 3a). LPS bioactivity correlated highly significantly also with the number of tender (*r*=+0.40 [95% CI +0.14 to 0.60], *p*=0.003) and swollen joints (*r*=+0.40 [95% CI +0.12 to 0.59], *p*=0.003). Furthermore, LPS bioactivity correlated significantly with patient-related outcomes (PROM) (Supplementary table 3a). LBP levels at 12 months also correlated with inflammatory parameters hsCRP (*r*=+0.54 [95% CI +0.31 to 0.71], *p*<0.001) and ESR (*r*=+0.45 [95% CI +0.20 to 0.64], *p*=0.001), as well as with DAS28-CRP (*r*=+0.37 [95% CI +0.11 to 0.58], *p*=0.005) and the number of swollen joints (*r*=+0.40 [95% CI +0.14 to 0.61], *p*=0.003), but not with tender joints (*r*=+0.22 [95% CI −0.06 to +0.46], *p*=0.11) (Supplementary table 3b). CD163 levels correlated with DAS28-CRP (*r*=+0.37 [95% CI +0.11 to 0.59], *p*=0.005) and ESR (*r*=+0.37 [95% CI +0.11 to 0.59], *p*=0.005), but not with CRP or number of swollen joints (Supplementary table 3c). CD14 levels correlated also with DAS28-CRP (*r*=+0.27 [95% CI +0.004 to 0.51], *p*=0.041) (Supplementary table 3d). In contrast, total serum LPS concentrations did not correlate significantly with most of the parameters of disease activity, inflammation, or LPS activity (Supplementary table 3e). Patients who failed to achieve ACR/EULAR remission after 12 months had higher levels of LPS bioactivity (mean±SD 0.22±0.09 vs 0.15±0.07 EU/ml, *p*<0.001), LBP (mean±SD 6985±2860 vs 5162±1715 ng/ml, *p*=0.008), and CD163 (mean±SD 1527±630 vs 1135±404 ng/ml, *p*=0.008) (Fig. 1a–c). Figure 2 and supplementary tables 3a-e present the correlations between all measured biomarkers and clinical characteristics.

### Serum LPS bioactivity and LBP levels at baseline correlate with disease activity at 12 months and the likelihood of achieving remission

As expected, disease activity decreased between baseline and 12-month follow-up visits (DAS28-CRP; mean±SD 3.54±1.09 vs 2.28±0.95, *p*<0.001). LPS bioactivity in the entire patient population, however, remained unchanged (Supplementary table 4). As the associations between LPS bioactivity and disease activity parameters became significant at 12 months, we explored the possibility that continuously elevated circulating LPS levels could relate to higher disease activity and also to less favorable treatment response. Indeed, LPS bioactivity measured at the baseline correlated significantly with disease activity at 12 months (DAS28-CRP, *r*=+0.29 [95% CI +0.02 to 0.52], *p*=0.031) as well as with ESR (*r*=+0.28 [95% CI +0.003 to 0.51], *p*=0.042), CRP (*r*=+0.30 [95% CI +0.03 to 0.53], *p*=0.025) and, in patients with early RA, with ACR/EULAR remission (mean±SD 0.24±0.09 EU/ml in ERA patients without remission vs 0.15±0.07 EU/ml, *p*=0.009; for all patients mean±SD 0.21±0.09 vs 0.17±0.07 EU/ml, *p*=0.065). In line with this, the level of LBP at baseline correlated significantly with disease activity (*r*=+0.34 [95% CI +0.07 to 0.56], *p*=0.012), hsCRP (*r*=+0.49 [95% CI +0.24 to 0.69], *p*<0.001) and swollen joints (*r*=+0.37 [95% CI +0.10 to 0.59], *p*=0.007) in the entire cohort at 12 months. Thus, high baseline levels of LPS bioactivity and LBP were both predictive of poor treatment response. Of the 20 patients with LPS bioactivity above median on both visits, only 3 (15%) reached ACR/EULAR remission at 12 months, whereas of the remaining 33 patients with LPS bioactivity below median, 18 (55%) had reached remission (*p*=0.004). Finally, LPS bioactivity at baseline correlated significantly with the levels of surrogate markers LBP (*r*=+0.31 [95% CI +0.03 to 0.54], *p*=0.025) and CD163 (*r*=+0.45 [95% CI +0.19 to 0.65], *p*<0.001) but not CD14 (*r*=+0.21 [95 % CI −0.08 to +0.46], *p*=0.14) at 12 months. Together these data suggest that levels of LPS bioactivity and the surrogate markers of LPS are relatively constant in RA patients, and the higher the levels are the less likely the patients are to reach remission.

**Fig. 3:**
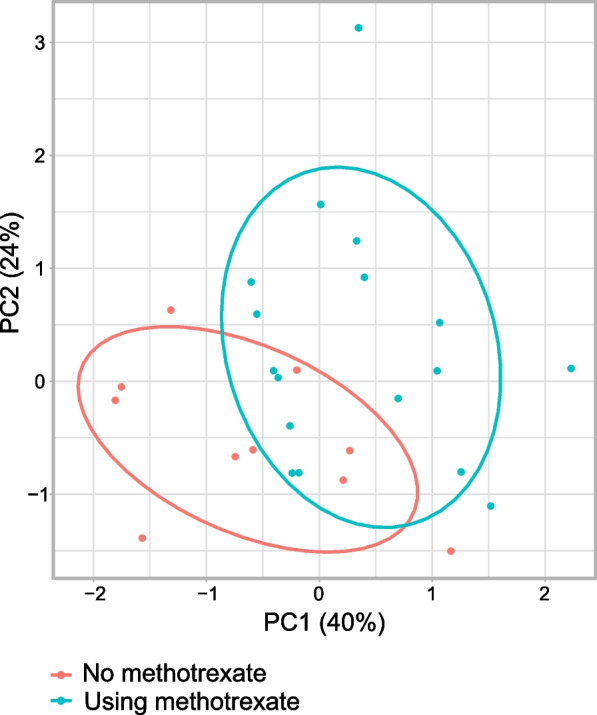
Principal component analysis of patients with chronic rheumatoid arthritis at the 12-month follow-up visit including LPS bioactivity and the concentrations of LBP, SAA, hsCRP, E-Selectin, YKL-40, and IL-6 in sera demonstrates clustering according to methotrexate use

We also attempted to study the effect of anti-rheumatic treatment on LPS bioactivity, but the small number of patients did not allow any detailed analysis. The use of Mtx was associated with lower LPS bioactivity and LBP level at 12 months in patients with CRA (mean±SD 0.26±0.11 vs 0.19±0.07 EU/ml, *p*=0.053 and 7811±2225 vs 5585±2077 ng/ml, *p*=0.016, respectively), suggesting that Mtx may in part modify disease activity by reducing systemic LPS bioactivity. Accordingly, in a principal component analysis of LPS bioactivity, LBP level, and inflammatory biomarkers in CRA patients of those on Mtx appeared to cluster apart from those who were not (Fig. 3).

### Neutralization of LPS abrogates the ability of serum to activate TLR4

Human serum contains various factors that potentially activate the TLR4 or NF-κB signaling, such as the acute phase proteins SAA, high mobility group box (HMGB) 1, and several cytokines. To explore the proportion of TLR4 activity contributed by serum LPS, we added polymyxin B to all serum samples to specifically neutralize LPS [27]. Polymyxin B abolished most of the TLR4 activity present in the sera of RA patients. No significant correlation existed between the residual TLR4 activity and parameters of inflammation, including SAA, or disease activity, except for the number of swollen joints at follow-up visit. In contrast, the proportion of TLR4 activity that was neutralized by polymyxin B correlated with the inflammatory parameters and disease activity in a similar manner to serum total TLR4 activity, suggesting that most of the TLR4 activity in the sera of RA patients is induced by LPS (Supplementary table 5a-b).

### LPS bioactivity, metabolic syndrome, and cardiovascular risk factors in RA patients

Metabolic endotoxemia is strongly associated with the metabolic syndrome, and obesity is associated with a less favorable prognosis of RA [28]. Patients with RA have an increased risk of cardiovascular diseases [29], and therefore, we explored the association of LPS bioactivity with cardiovascular risk factors, although several risk factors such as diabetes had been excluded from the patient cohort. LPS bioactivity was associated with higher blood pressure both at baseline (*r*=+0.40 [95% CI +0.14 to 0.61], *p*=0.002 for systolic and *r*=+0.37 [95% CI +0.11 to 0.59], *p*=0.005 for diastolic blood pressure) and at 12 months (*r*=+0.26 [95% CI −0.01 to 0.50], *p*=0.055 for systolic and *r*=+0.36 [95% CI +0.10 to 0.58], *p*=0.007 for diastolic blood pressure). As expected, LPS bioactivity associated with increased BMI both at baseline (*r*=+0.42 [95% CI +0.17 to 0.62], *p*=0.002) and at the 12-month visit (*r*=+0.39 [95% CI +0.14 to 0.60], *p*=0.003) (Fig. 2). The proportion of adipose tissue correlated significantly with LPS bioactivity (at baseline *r*=+0.47 [95% CI +0.21 to 0.67], *p*=0.001; at 12 months *r*=+0.36 [95% CI +0.09 to 0.58], *p*=0.007) as did also CD163 levels (BMI: *r*=+0.26 [95% CI −0.02 to 0.50], *p*=0.061 at baseline, *r*=+0.45 [95% CI +0.21 to 0.64], *p*<0.001 at 12 months; adipose tissue: *r*=+0.35 [95% CI +0.07 to 0.58], *p*=0.014 at baseline, *r*=+0.43 [95% CI +0.17 to 0.63], *p*=0.001 at 12 months) (Fig. 2). High BMI at baseline also associated with a decreased likelihood of achieving ACR/EULAR remission at 12 months (mean±SD 25.8±4.2 vs 22.7±3.3, *p*=0.003). HbA1c levels correlated with LBP at the follow-up visit (*r*=+0.37 [95% CI +0.11 to 0.58], *p*=0.005). HbA1c also correlated with DAS28-CRP at 12 months (*r*=+0.31 [+0.05 to 0.53], *p*=0.019), consistent with the possibility that hyperglycemia can disturb the intestinal barrier function [30].

## Discussion

In this study, we demonstrate that the LPS bioactivity of the sera of RA patients correlates with inflammatory parameters, disease activity, and patient-reported outcome measures (PROMs). Importantly, LPS bioactivity measured at baseline correlated with disease activity at 12-month follow-up and predicted a decreased likelihood of remission. No correlation existed between LPS bioactivity and disease activity at baseline, however. At baseline, the patients had either not used any DMARDs or their response to DMARD treatment had been inadequate, and therefore, they had high disease activity probably also due to factors other than LPS, which may have masked the less robust pro-inflammatory effect of LPS. Overall LPS bioactivity did not change significantly between baseline and 12-month visits, which raises the possibility that serum LPS bioactivity is an independent patient-related factor, which predicts higher disease activity and manifests as a poor response to anti-rheumatic treatment. Sustained high LPS bioactivity at both visits was indeed associated with a lower probability of remission.

Measuring LPS levels from complex biological samples such as serum is technically challenging [19, 31]. In addition, the biological activity of LPS derived from different bacterial species varies significantly, and the total serum LPS level may thus not reflect their actual pro-inflammatory impact on cells [20, 21]. This is supported by our current findings, but also by the lack of studies demonstrating correlation between serum LPS level and RA disease activity. In contrast to LPS levels, several studies have found that surrogate markers of LPS, LBP in particular, correlate with disease activity, and LBP has been suggested to serve as a biomarker of RA activity [32, 33]. LBP is, however, an acute phase protein induced also by factors other than LPS, such as IL-1β, IL-6, and tumor necrosis factor, and as such, it is a non-specific indicator for LPS [34]. In our cohort, TLR4 activation representing LPS bioactivity correlated with the PROMs better than LBP, but more significant correlations were observed between LBP and the inflammatory parameters. Thus, TLR4 activation may reflect more accurately the total bioactivity of different LPS varieties present in sera than the surrogate markers. TLR4 activity as a measure of serum LPS bioactivity has been previously studied in healthy volunteers. Thaiss et al. [30] found a correlation between serum TLR4 activation and the levels of HbA1c and systolic blood pressure. Factors other than LPS, such as SAA or HMGB1, can also activate the TLR4. Therefore, we specifically neutralized LPS by polymyxin B [27]. Addition of polymyxin B abolished most of the TLR4 activity, strongly suggesting that the measured serum TLR4 activity was mainly contributed by the LPS.

The cause of higher LPS bioactivity in RA patients with active disease is unclear. RA patients have been shown to have intestinal dysbiosis, which could increase circulating LPS levels or promote the release of more pro-inflammatory LPS types with higher capability to activate TLR4 signaling [35, 36]. Increased intestinal permeability has also been described in RA patients [37]. Dysfunctions in the mechanisms that neutralize or detoxify LPS molecules, such as dephosphorylation of the lipid A moiety by intestinal alkaline phosphatase or transfer of LPS to lipoprotein particles could be involved [38, 39]. However, it should be noted that our findings do not implicate that the LPS levels in RA patients would in general be higher than in healthy controls, but only that, in RA patients, higher TLR4 activity is associated with higher disease activity.

Pharmacological or dietary manipulation of LPS-induced inflammatory signaling could present an attractive target for drug development, and the present results would support such an approach [40, 41]. A recent phase II clinical trial of a monoclonal TLR4-blocking antibody failed to demonstrate significant clinical efficacy compared to Mtx alone [42]. However, in addition to TLR4, LPS activates TLR2 and also both the non-canonical caspase 4/5 inflammasome and the so-called atypical inflammasome [43, 44]. Therefore, inhibiting only TLR4 may be inadequate to eliminate the pro-inflammatory effects of LPS. Mtx modulates gut microbiota, which may also in part explain the lack of difference between treatment groups [45]. An alternative approach could be to attempt to reduce the intestinal permeability.

The limitations of this study include the moderate number of participants and the lack of healthy controls. Only limited conclusions could be made regarding the effects of LPS on cardiovascular disease risk parameters in RA patients due to the exclusion criteria.

In conclusion, we demonstrate—to our knowledge for the first time—that in RA patients the serum LPS bioactivity associates significantly with disease activity and correlates with surrogate markers of LPS, in particular with LBP. Furthermore, serum LPS bioactivity is an independent patient-related factor, which associates with disease activity and predicts a decreased likelihood of achieving remission.

## Supplementary Information

**Additional file 1: Supplementary table 1.** Patient cohort characteristics at baseline.

**Additional file 2: Supplementary table 2.** The number of patients with missing data.

**Additional file 3: Supplementary table 3a-e.** Correlations of LPS-related biomarkers with RA disease activity, inflammatory biomarkers, and metabolic factors.

**Additional file 4: Supplementary table 4.** Measured LPS bioactivity and the concentrations of LBP, CD14, and CD163.

**Additional file 5: Supplementary table 5a-b.** Serum TLR4 activity and LPS bioactivity determined after neutralization by polymyxin B.

## Data Availability

The dataset supporting the conclusions of this article is available from the corresponding author upon reasonable request.

## Abbreviations

RA: Rheumatoid arthritis
LPS: Lipopolysaccharide
TLR: Toll-like receptor
CD: Cluster of differentiation
ELISA: Enzyme-linked immunosorbent assay
DAS: Disease activity score
(hs)CRP: (High-sensitivity) C-reactive protein
PAMP: Pathogen-associated molecular pattern
ACPA: Anti-citrullinated protein autoantibody
NF: Nuclear factor
LBP: Lipopolysaccharide-binding protein
MD: Myeloid differentiation
EULAR: European Alliance of Associations for Rheumatology
ACR: American College of Rheumatology
ERA: Early rheumatoid arthritis
(cs)DMARD: (Conventional synthetic) disease-modifying anti-rheumatic drug
CRA: Chronic rheumatoid arthritis
HAQ: Health assessment questionnaire
DAI: Disease Activity Index
SEAP: Secreted alkaline phosphatase
OD: Optical density
SAA: Serum amyloid A
IL: Interleukin
HDL: High-density lipoprotein
LDL: Low-density lipoprotein
TG: Triglyceride
BMI: Body mass index
CI: Confidence interval
ESR: Erythrocyte sedimentation rate
SD: Standard deviation
Mtx: Methotrexate
HMGB: High mobility group box
PROM: Patient-reported outcome measure
