# Supplementary material for: The biological activity of serum bacterial lipopolysaccharides associates with disease activity and likelihood of achieving remission in patients with rheumatoid arthritis
Source: Arthritis Res Ther. 2022 Nov 21;24:256. doi: 10.1186/s13075-022-02946-z (PMC9677706; doi:10.1186/s13075-022-02946-z)
Supplement: Supplementary file 1 — Additional file 1: Supplementary table 1. Patient cohort characteristics at baseline. [file 13075_2022_2946_MOESM1_ESM.pdf]

Supplementary table 1. Patient cohort characteristics at baseline.

	Early RA (n = 30)		Chronic RA (n = 28)		All patients (n = 58)	
Age (years)	49	(14)	48	(13)	49	(14)
Disease duration (months)	10	(14)	187	(133)	92	(127)
Body mass index	24	(4)	26	(4)	25	(4)
Reformed smoker	13	(43)	6	(21)	19	(33)
Metabolic syndrome	7	(23)	5	(18)	12	(21)
Body fat (%)	29	(11)	32	(10)	31	(11)
Systolic blood pressure (mmHg)	138	(19)	141	(24)	139	(22)
Diastolic blood pressure (mmHg)	84	(10)	87	(13)	85	(12)
Rheumatic factor present	26	(87)	22	(79)	48	(83)
Antibodies against CCP present	27	(90)	24	(86)	51	(88)
Swollen joint count*	5	(4)	3	(4)	4	(4)
Tender joint count*	4	(4)	2	(3)	3	(3)
DAS28-CRP	3.7	(1.0)	3.3	(1.1)	3.5	(1.1)
Pain (VAS)	43	(28)	48	(25)	46	(26)
Patient global assessment (VAS)	39	(24)	44	(26)	41	(25)
Health Assessment Questionnaire score	0.5	(0.4)	0.9	(0.7)	0.7	(0.6)
Erythrocyte sedimentation rate (mm/h)	21	(17)	18	(18)	20	(17)
CRP (highly sensitive, mg/l)	11	(15)	7	(7)	9	(12)
Serum Amyloid A (mg/ml)	160	(239)	243	(335)	201	(291)
E-Selectin (ng/ml)	28	(11)	26	(10)	27	(11)
Resistin (ng/ml)	16	(4)	15	(5)	16	(5)
Visfatin (ng/ml)	10	(9)	10	(6)	10	(8)
YKL-40 (ng/ml)	70	(33)	84	(55)	77	(45)
IL-6 (pg/ml)	9	(10)	9	(13)	9	(11)
Total cholesterol (mmol/l)	4.9	(1.0)	5.2	(1.0)	5.0	(1.0)
LDL cholesterol (mmol/l)	3.0	(0.8)	3.0	(0.8)	3.0	(0.8)
HDL cholesterol (mmol/l)	1.7	(0.5)	2.0	(0.4)	1.8	(0.5)
Triglyceride concentration (mmol/l)	0.9	(0.3)	0.9	(0.3)	0.9	(0.3)
HbA1c (mmol/mol)	37	(3)	34	(3)	35	(3)

Presented as n (%) or mean (SD).

*28 joints were evaluated.

RA, rheumatoid arthritis; SD, standard deviation; CCP, Cyclic citrullinated peptide; DAS, Disease Activity Score; CRP, C-reactive protein; LDL, Low-Density lipoprotein; HDL, High-density lipoprotein; IL-6, Interleukin 6; VAS, Visual analog scale.
