# Supplementary material for: The biological activity of serum bacterial lipopolysaccharides associates with disease activity and likelihood of achieving remission in patients with rheumatoid arthritis
Source: Arthritis Res Ther. 2022 Nov 21;24:256. doi: 10.1186/s13075-022-02946-z (PMC9677706; doi:10.1186/s13075-022-02946-z)
Supplement: Supplementary file 2 — Additional file 2: Supplementary table 2. The number of patients with missing data. [file 13075_2022_2946_MOESM2_ESM.pdf]

Supplementary table 5. The number of patients (n=58) with missing data at baseline and follow-up visits.

	Baseline	Follow-up
Disease duration	2 (3)	2 (3)
Body mass index	1 (2)	0
Body fat	6 (10)	1 (2)
Blood pressure	0	1 (2)
Antibodies against CCP present	1 (2)	1 (2)
Swollen joint count	0	1 (2)
Tender joint count	1 (2)	1 (2)
DAS28-CRP	3 (5)	0
Pain (visual analog scale)	2 (3)	0 (3)
Global health (visual analog scale)	1 (2)	0
Health assessment questionnaire score	2 (3)	0 (3)
Erythrocyte sedimentation rate	0	1 (2)
CRP, highly sensitive	1 (2)	3 (5)
Serum amyloid A	1 (2)	0
E-selectin	2 (3)	2 (3)
YKL-40	2 (3)	2 (3)
IL-6	2 (3)	2 (3)
Total cholesterol	1 (2)	0
HbA1c	3 (5)	0
LPS bioactivity	3 (5)	2 (3)
LPS-binding protein	4 (7)	2 (3)
CD163	3 (5)	2 (3)
LPS concentration (EndoLISA)	4 (7)	2 (3)

Reported as n (%). Variables with no missing data are omitted.
DAS, Disease activity score; CRP, C-reactive protein; IL, Interleukin;
LPS, Lipopolysaccharide; CD, Cluster of differentiation.
