# Supplementary material for: The biological activity of serum bacterial lipopolysaccharides associates with disease activity and likelihood of achieving remission in patients with rheumatoid arthritis
Source: Arthritis Res Ther. 2022 Nov 21;24:256. doi: 10.1186/s13075-022-02946-z (PMC9677706; doi:10.1186/s13075-022-02946-z)
Supplement: Supplementary file 3 — Additional file 3: Supplementary table 3a-e. Correlations of LPS-related biomarkers with RA disease activity, inflammatory biomarkers, and metabolic factors. [file 13075_2022_2946_MOESM3_ESM.pdf]

Supplementary table 3a. Correlations of serum LPS bioactivity with RA disease activity, inflammatory biomarkers, and metabolic factors.

| | Baseline | | | Follow-up | | |
|---|---|---|---|---|---|---|
| | p | r* | 95% CI | p | r* | 95% CI |
| LBP | | | | 0.017 | +0.32 | +0.05 to 0.54 |
| CD163 | <0.001 | +0.44 | +0.19 to 0.63 | 0.008 | +0.35 | +0.09 to 0.57 |
| CD14 | | | | | | |
| LPS (EndoLISA) | | | | | | |
| | | | | | | |
| Age | <0.001 | +0.45 | +0.21 to 0.64 | 0.003 | +0.39 | +0.14 to 0.60 |
| DAS28-CRP | | | | <0.001 | +0.48 | +0.24 to 0.67 |
| Swollen joints (DAS28) | | | | 0.004 | +0.38 | +0.12 to 0.59 |
| Tender joints (DAS28) | | | | 0.003 | +0.40 | +0.14 to 0.60 |
| Patient global assessment (VAS) | | | | 0.001 | +0.42 | +0.17 to 0.62 |
| Pain (VAS) | | | | 0.001 | +0.44 | +0.19 to 0.63 |
| HAQ | | | | 0.001 | +0.44 | +0.19 to 0.63 |
| | | | | | | |
| hsCRP | | | | 0.002 | +0.41 | +0.15 to 0.61 |
| ESR | 0.037 | +0.28 | +0.01 to 0.52 | 0.030 | +0.29 | +0.02 to 0.52 |
| Serum amyloid A | 0.008 | +0.35 | +0.09 to 0.57 | 0.001 | +0.42 | +0.17 to 0.62 |
| YKL-40 | 0.042 | +0.28 | +0.002 to 0.51 | <0.001 | +0.50 | +0.26 to 0.68 |
| E-Selectin | 0.013 | +0.34 | +0.07 to 0.56 | 0.001 | +0.42 | +0.17 to 0.62 |
| Resistin | | | | 0.014 | +0.33 | +0.06 to 0.55 |
| Visfatin | | | | | | |
| IL-6 | | | | <0.001 | +0.43 | +0.18 to 0.63 |
| | | | | | | |
| Body mass index | 0.002 | +0.42 | +0.17 to 0.62 | 0.003 | +0.39 | +0.14 to 0.60 |
| Adipose tissue | 0.001 | +0.47 | +0.21 to 0.67 | 0.007 | +0.36 | +0.09 to 0.58 |
| Total cholesterol | | | | 0.029 | +0.29 | +0.02 to 0.52 |
| HDL cholesterol | | | | | | |
| LDL cholesterol | | | | | | |
| Triglycerides | | | | | | |
| HbA1c | 0.024 | +0.31 | +0.04 to 0.55 | | | |
| Systolic blood pressure | 0.002 | +0.40 | +0.14 to 0.61 | | | |
| Diastolic blood pressure | 0.005 | +0.37 | +0.11 to 0.58 | 0.007 | +0.36 | +0.10 to 0.58 |

Statistically non-significant correlations are omitted.
* Spearman correlation coefficient
LPS, Lipopolysaccharide; RA, Rheumatoid arthritis; LBP, LPS-Binding Protein; CD, Cluster of differentiation; hsCRP, high-sensitivity C-reactive protein; DAS, Disease activity score; VAS, Visual analog scale; HAQ, Health assessment questionnaire; ESR, Erythrocyte sedimentation rate; IL, Interleukin; HDL, High-density lipoprotein; LDL, Low-density lipoprotein; CI, Confidence interval.

Supplementary table 3b. Correlations of serum LBP concentration with RA disease activity, inflammatory biomarkers, and metabolic factors.

| | Baseline | | | Follow-up | | |
|---|---|---|---|---|---|---|
| | p | r* | 95% CI | p | r* | 95% CI |
| LPS Bioactivity | | | | 0.017 | +0.32 | +0.05 to 0.54 |
| CD163 | <0.001 | +0.50 | +0.26 to 0.68 | 0.028 | +0.29 | +0.03 to 0.52 |
| CD14 | 0.002 | +0.42 | +0.16 to 0.62 | <0.001 | +0.51 | +0.28 to 0.69 |
| LPS (EndoLISA) | | | | | | |
| | | | | | | |
| Age | | | | | | |
| DAS28-CRP | 0.004 | +0.39 | +0.13 to 0.60 | 0.005 | +0.37 | +0.11 to 0.58 |
| Swollen joints (DAS28) | 0.035 | +0.29 | +0.01 to 0.52 | 0.003 | +0.40 | +0.14 to 0.61 |
| Tender joints (DAS28) | | | | | | |
| Patient global assessment (VAS) | | | | 0.011 | +0.34 | +0.07 to 0.56 |
| Pain (VAS) | | | | 0.005 | +0.37 | +0.11 to 0.58 |
| HAQ | | | | | | |
| | | | | | | |
| hsCRP | <0.001 | +0.65 | +0.45 to 0.78 | <0.001 | +0.54 | +0.31 to 0.71 |
| ESR | <0.001 | +0.47 | +0.22 to 0.66 | 0.001 | +0.45 | +0.20 to 0.64 |
| Serum amyloid A | <0.001 | +0.47 | +0.43 to 0.77 | <0.001 | +0.47 | +0.23 to 0.66 |
| YKL-40 | | | | 0.003 | +0.39 | +0.13 to 0.59 |
| E-Selectin | 0.030 | +0.30 | +0.02 to 0.53 | | | |
| Resistin | | | | | | |
| Visfatin | | | | | | |
| IL-6 | 0.001 | +0.45 | +0.20 to 0.64 | 0.002 | +0.40 | +0.15 to 0.61 |
| | | | | | | |
| Body mass index | | | | | | |
| Adipose tissue | | | | | | |
| Total cholesterol | | | | | | |
| HDL cholesterol | | | | | | |
| LDL cholesterol | | | | | | |
| Triglycerides | | | | | | |
| HbA1c | | | | 0.005 | +0.37 | +0.11 to 0.58 |
| Systolic blood pressure | | | | 0.015 | +0.33 | +0.06 to 0.55 |
| Diastolic blood pressure | | | | | | |

Supplementary table 3c. Correlations of serum CD163 concentration with RA disease activity, inflammatory biomarkers, and metabolic factors.

| | Baseline | | | Follow-up | | |
|---|---|---|---|---|---|---|
| | p | r* | 95% CI | p | r* | 95% CI |
| LPS Bioactivity | <0.001 | +0.44 | +0.19 to 0.63 | 0.008 | +0.35 | +0.09 to 0.57 |
| LBP | <0.001 | +0.50 | +0.26 to 0.68 | 0.028 | +0.29 | +0.03 to 0.52 |
| CD14 | 0.002 | +0.41 | +0.16 to 0.62 | 0.003 | +0.39 | +0.13 to 0.60 |
| LPS (EndoLISA) | <0.001 | +0.44 | +0.19 to 0.64 | | | |
| | | | | | | |
| Age | 0.003 | +0.39 | +0.13 to 0.60 | <0.001 | +0.46 | +0.21 to 0.65 |
| DAS28-CRP | <0.001 | +0.50 | +0.26 to 0.68 | 0.005 | +0.37 | +0.11 to 0.58 |
| Swollen joints (DAS28) | 0.002 | +0.41 | +0.16 to 0.62 | | | |
| Tender joints (DAS28) | 0.003 | +0.39 | +0.13 to 0.60 | 0.023 | +0.31 | +0.04 to 0.54 |
| Patient global assessment (VAS) | | | | 0.007 | +0.36 | +0.09 to 0.57 |
| Pain (VAS) | | | | 0.006 | +0.36 | +0.10 to 0.58 |
| HAQ | | | | <0.001 | +0.47 | +0.23 to 0.66 |
| | | | | | | |
| hsCRP | 0.004 | +0.38 | +0.12 to 0.59 | | | |
| ESR | 0.003 | +0.39 | +0.13 to 0.60 | 0.005 | +0.37 | +0.11 to 0.59 |
| Serum amyloid A | 0.001 | +0.43 | +0.18 to 0.63 | 0.023 | +0.31 | +0.04 to 0.53 |
| YKL-40 | | | | 0.006 | +0.36 | +0.10 to 0.58 |
| E-Selectin | | | | 0.007 | +0.36 | +0.10 to 0.57 |
| Resistin | | | | | | |
| Visfatin | 0.039 | +0.28 | +0.007 to 0.51 | | | |
| IL-6 | 0.034 | +0.29 | +0.02 to 0.52 | <0.001 | +0.46 | +0.22 to 0.65 |
| | | | | | | |
| Body mass index | | | | <0.001 | +0.45 | +0.21 to 0.64 |
| Adipose tissue | 0.014 | +0.35 | +0.07 to 0.57 | 0.001 | +0.43 | +0.17 to 0.63 |
| Total cholesterol | | | | | | |
| HDL cholesterol | 0.034 | -0.29 | -0.52 to -0.02 | | | |
| LDL cholesterol | | | | 0.022 | +0.31 | +0.04 to 0.53 |
| Triglycerides | | | | 0.048 | +0.27 | -0.006 to 0.50 |
| HbA1c | | | | | | |
| Systolic blood pressure | | | | | | |
| Diastolic blood pressure | | | | | | |

Supplementary table 3d. Correlations of serum CD14 concentration with RA disease activity, inflammatory biomarkers, and metabolic factors.

| | Baseline | | | Follow-up | | |
|---|---|---|---|---|---|---|
| | p | r* | 95% CI | p | r* | 95% CI |
| LPS Bioactivity | | | | | | |
| LBP | 0.002 | +0.42 | +0.16 to 0.62 | <0.001 | +0.51 | +0.28 to 0.69 |
| CD163 | 0.002 | +0.41 | +0.16 to 0.62 | 0.003 | +0.39 | +0.13 to 0.60 |
| LPS (EndoLISA) | 0.005 | +0.38 | +0.12 to 0.59 | | | |
| | | | | | | |
| Age | | | | 0.014 | +0.33 | +0.06 to 0.55 |
| DAS28-CRP | | | | 0.041 | +0.27 | +0.004 to 0.51 |
| Swollen joints (DAS28) | | | | | | |
| Tender joints (DAS28) | | | | | | |
| Patient global assessment (VAS) | | | | | | |
| Pain (VAS) | | | | | | |
| HAQ | 0.038 | +0.29 | +0.009 to 0.52 | | | |
| | | | | | | |
| hsCRP | | | | | | |
| ESR | 0.002 | +0.42 | +0.16 to 0.62 | 0.001 | +0.43 | +0.17 to 0.53 |
| Serum amyloid A | 0.018 | +0.32 | +0.05 to 0.54 | 0.002 | +0.41 | +0.15 to 0.61 |
| YKL-40 | | | | 0.003 | +0.39 | +0.14 to 0.60 |
| E-Selectin | | | | | | |
| Resistin | | | | | | |
| Visfatin | | | | | | |
| IL-6 | | | | 0.012 | +0.33 | +0.07 to 0.55 |
| | | | | | | |
| Body mass index | | | | | | |
| Adipose tissue | | | | | | |
| Total cholesterol | 0.014 | +0.33 | +0.07 to 0.56 | <0.001 | +0.47 | +0.23 to 0.66 |
| HDL cholesterol | | | | | | |
| LDL cholesterol | 0.030 | +0.29 | +0.02 to 0.52 | <0.001 | +0.47 | +0.23 to 0.66 |
| Triglycerides | | | | | | |
| HbA1c | | | | | | |
| Systolic blood pressure | | | | 0.043 | +0.27 | +0.001 to 0.51 |
| Diastolic blood pressure | | | | | | |

Supplementary table 3e. Correlations of serum LPS concentration (EndoLISA) with RA disease activity, inflammatory biomarkers, and metabolic factors.

| | Baseline | | | Follow-up | | |
|---|---|---|---|---|---|---|
| | p | r* | 95% CI | p | r* | 95% CI |
| LPS Bioactivity | | | | | | |
| LBP | | | | | | |
| CD163 | 0.001 | +0.44 | +0.19 to 0.64 | | | |
| CD14 | 0.005 | +0.38 | +0.12 to 0.59 | | | |
| | | | | | | |
| Age | 0.010 | +0.35 | +0.08 to 0.57 | 0.018 | +0.32 | +0.05 to 0.54 |
| DAS28-CRP | | | | | | |
| Swollen joints (DAS28) | | | | | | |
| Tender joints (DAS28) | | | | | | |
| Patient global assessment (VAS) | | | | | | |
| Pain (VAS) | | | | | | |
| HAQ | | | | | | |
| | | | | | | |
| hsCRP | | | | | | |
| ESR | | | | | | |
| Serum amyloid A | | | | | | |
| YKL-40 | | | | 0.006 | +0.36 | +0.10 to 0.58 |
| E-Selectin | | | | | | |
| Resistin | | | | | | |
| Visfatin | | | | | | |
| IL-6 | | | | | | |
| | | | | | | |
| Body mass index | | | | | | |
| Adipose tissue | | | | | | |
| Total cholesterol | 0.023 | +0.31 | +0.04 to 0.54 | | | |
| HDL cholesterol | | | | | | |
| LDL cholesterol | 0.013 | +0.34 | +0.07 to 0.56 | | | |
| Triglycerides | | | | | | |
| HbA1c | | | | | | |
| Systolic blood pressure | | | | | | |
| Diastolic blood pressure | | | | | | |
