# Supplementary material for: The biological activity of serum bacterial lipopolysaccharides associates with disease activity and likelihood of achieving remission in patients with rheumatoid arthritis
Source: Arthritis Res Ther. 2022 Nov 21;24:256. doi: 10.1186/s13075-022-02946-z (PMC9677706; doi:10.1186/s13075-022-02946-z)
Supplement: Supplementary file 4 — Additional file 4: Supplementary table 4. Measured LPS bioactivity and the concentrations of LBP, CD14, and CD163. [file 13075_2022_2946_MOESM4_ESM.pdf]

Supplementary table 4. Measured LPS bioactivity and the concentrations of LBP, CD14, and CD163.

	ERA (n=30)			CRA (n=28)			All patients (n=58)		
LPS bioactivity (EU/ml)	mean		SD	mean		SD	mean		SD
baseline	0.185		0.086	0.205		0.090	0.195		0.088
follow-up	0.174		0.078	0.215		0.096	0.194		0.088
LBP (ng/ml)									
baseline	8602		3615	8218		4019	8410		3791
follow-up	6138	***	2866	6409	*	2360	6269	***	2614
CD14 (ng/ml)									
baseline	2089		694	2139		775	2114		728
follow-up	1998		480	1972		463	1985		468
CD163 (ng/ml)									
baseline	1884		773	1528	†	657	1709		734
follow-up	1325	***	520	1423		645	1372	***	580

*** $p \leq 0.001$, ** $p \leq 0.01$, * $p \leq 0.05$ for difference between baseline and follow-up measurements.

† $p \leq 0.05$ for difference between ERA vs CRA.

SD, Standard Deviation; LPS, lipopolysaccharide; ERA, Early Rheumatoid Arthritis; CRA, Chronic rheumatoid arthritis; LBP, LPS-Binding Protein; CD, Cluster of differentiation.
