# Supplementary material for: The biological activity of serum bacterial lipopolysaccharides associates with disease activity and likelihood of achieving remission in patients with rheumatoid arthritis
Source: Arthritis Res Ther. 2022 Nov 21;24:256. doi: 10.1186/s13075-022-02946-z (PMC9677706; doi:10.1186/s13075-022-02946-z)
Supplement: Supplementary file 5 — Additional file 5: Supplementary table 5a-b. Serum TLR4 activity and LPS bioactivity determined after neutralization by polymyxin B. [file 13075_2022_2946_MOESM5_ESM.pdf]

Supplementary table 5a. Serum TLR4 activity and LPS bioactivity determined after neutralization by polymyxin B and correlations with RA disease activity and inflammatory biomarkers at baseline.

	mean	SD
Total TLR4 †	0.276	0.182
LPS†	0.117	0.106
Residual TLR4 †	0.158	0.130

	Total TLR4			LPS			Residual TLR4		
	p	r§	95% CI	p	r§	95% CI	p	r§	95% CI
Age									
Body mass index	0.034	+0.29	+0.02 to 0.52				0.040	+0.28	+0.005 to 0.52
DAS28-CRP									
SJC (DAS28)									
TJC (DAS28)									
PGA (VAS)									
Pain (VAS)									
HAQ									
hsCRP									
ESR									
Serum amyloid A									
YKL-40									
E-selectin				0.037	+0.28	+0.10 to 0.52			
Resistin									
Visfatin									
IL-6									

Statistically non-significant correlations are omitted.

§ Spearman correlation coefficient. † EU (Endotoxin unit)/ml.

SD, Standard deviation; LPS, Lipopolysaccharide; RA, Rheumatoid arthritis; VAS, Visual analog scale; DAS, Disease activity score; TLR, Toll-like receptor; hsCRP, high-sensitivity C-reactive protein; ESR, Erythrocyte sedimentation rate; IL, Interleukin; HAQ, Health assessment questionnaire; PGA, Patient global assessment; SJC, Swollen joint count; TJC, Tender joint count.

Supplementary table 5b. Serum TLR4 activity and LPS bioactivity determined after neutralization by polymyxin B and correlations with RA disease activity and inflammatory biomarkers at follow-up.

	mean		SD
Total TLR4 †	0.224	**	0.135
LPS†	0.084	*	0.066
Residual TLR4 †	0.140		0.120

	**Total TLR4**			**LPS**			**Residual TLR4**		
	p	r§	95% CI	p	r§	95% CI	p	r§	95% CI
Age	0.008	+0.35	+0.09 to 0.57	0.036	+0.28	+0.01 to 0.51			
Body mass index	0.028	+0.29	+0.03 to 0.52	0.002	+0.41	+0.16 to 0.61			
DAS28-CRP	0.006	+0.36	+0.10 to 0.58	0.007	+0.35	+0.09 to 0.57			
SJC (DAS28)	0.015	+0.33	+0.06 to 0.55				0.020	+0.31	+0.04 to 0.54
TJC (DAS28)	0.023	+0.31	+0.04 to 0.53	0.024	+0.31	+0.04 to 0.53			
PGA (VAS)	0.020	+0.31	+0.04 to 0.54	0.017	+0.32	+0.05 to 0.54			
Pain (VAS)									
HAQ	0.046	+0.27	-0.003 to 0.50						
hsCRP				0.008	+0.36	+0.09 to 0.57			
ESR									
Serum amyloid A				0.020	+0.31	+0.04 to 0.54			
YKL-40									
E-selectin				0.028	+0.29	+0.03 to 0.52			
Resistin									
Visfatin									
IL-6				0.015	+0.32	+0.06 to 0.55			

** p<0.01, * p<0.05 between baseline and follow-up visits.
